# Electrospun Biomimetic Multifunctional Nanofibers Loaded with Ferulic Acid for Enhanced Antimicrobial and Wound-Healing Activities in STZ-Induced Diabetic Rats

**DOI:** 10.3390/ph15030302

**Published:** 2022-02-28

**Authors:** Sneha Anand, Prashant Pandey, Mohammed Yasmin Begum, Kumarappan Chidambaram, Dilip Kumar Arya, Ravi Kr. Gupta, Ruchi Sankhwar, Shweta Jaiswal, Sunita Thakur, Paruvathanahalli Siddalingam Rajinikanth

**Affiliations:** 1 Department of Pharmaceutical Sciences, Babasaheb Bhimrao Ambedkar University, Lucknow 226025, India; snehaanand.rs@bbau.ac.in (S.A.); prashant.p@bbau.ac.in (P.P.); dkarya.rs@bbau.ac.in (D.K.A.); shwetajaiswal.rs@bbau.ac.in (S.J.); sunitathakurbbau@gmail.com (S.T.); 2 Department of Pharmaceutics, King Khalid University, Abha 62529, Saudi Arabia; ybajen@kku.edu.sa; 3 Department of Pharmacology & Toxicology, King Khalid University, Abha 62529, Saudi Arabia; kumarappan@kku.edu.sa; 4 Department of Environmental Microbiology, Babasaheb Bhimrao Ambedkar University, Lucknow 226025, India; ravi.gu81@bbau.ac.in (R.K.G.); ruchi.rs@email.bbau.ac.in (R.S.)

**Keywords:** silk sericin, ferulic acid, electrospinning, polycaprolactone, cellulose acetate, diabetic foot ulcer, diabetic wound repair

## Abstract

Diabetic foot ulceration is the most distressing complication of diabetes having no standard therapy. Nanofibers are an emerging and versatile nanotechnology-based drug-delivery system with unique wound-healing properties. This study aimed to prepare and evaluate silk-sericin based hybrid nanofibrous mats for diabetic foot ulcer. The nanofibrous mats were prepared by electrospinning using silk sericin mixed with different proportions of polycaprolactone (PCL) and cellulose acetate (CA) loaded with ferulic acid (FA). The in vitro characterizations, such as surface morphology, mechanical properties, swelling behavior, biodegradability, scanning electron microscopy, and drug release were carried out. The SEM images indicated that nanofibers formed with varied diameters, ranging from 100 to 250 nm, and their tensile strength was found to range from 7 to 15 MPa. In vitro release demonstrated that the nanofibers sustained FA release over an extended time of period. In vitro cytotoxicity showed that the nanofibers possessed a lower cytotoxicity in HaCaT cells. The in vivo wound-healing studies demonstrated an excellent wound-healing efficiency of the nanofibers in diabetic rats. Furthermore, the histopathological studies showed the nanofibers’ ability to restore the skin’s normal structure. Therefore, it was concluded that the prepared silk-sericin-based hybrid nanofibers loaded with FA could be a promising drug-delivery platform for the effective treatment of diabetic foot ulcers.

## 1. Introduction

Diabetic foot ulceration is the most distressing complication of diabetes, which significantly impacts a patient’s health, quality of life, and life expectancy [1]. Nonhealing foot ulcers result in significant functional impairment, reduced quality of life, and a substantial economic burden to patients and the healthcare system, even though they are preventable [2]. Nowadays, many new wound-healing products have entered the market that aid in wound healing in diabetes by reducing the wound area and improving the rates of limb healing. Unfortunately, they are ineffective in controlling and healing diabetic foot ulcers because they cannot be quickly absorbed, cannot provide an ideal moist environment, cannot provide drugs with a sustained and effective release, and cannot control biofilm formation at the wound site [3]. Therefore, there are challenges associated with advancing novel drug-delivery systems to address the unmet medical need for better treatment of diabetic foot ulcers [4]. Electrospun-nanofiber wound dressings have shown a multifunctional capability in healing wounds, including diabetic foot ulcers, by providing rapid absorption, maintaining the moisture microenvironment, promoting better oxygenation, promoting angiogenesis, and providing sustained and controlled release of therapeutic agents at the wound site to control the formation of biofilms. Additionally, nanofibers having a diameter the same as the extracellular matrix (ECM) have been proven to accelerate cellular processes such as cell adhesion, attachment, migration into the wound site, and proliferation [5,6,7,8].

Ferulic acid (FA) is a phenolic compound and a natural antioxidant with extensive therapeutic activities. It has physiological properties such as anti-inflammatory, anticancer, antimicrobial, and antidiabetic [9]. FA’s benefits are limited due to its poor solubility and bioavailability [10,11]. Electrospinning has been highlighted in the growing popularity and advancement of pharmaceuticals and nanotechnology for its ability to fabricate high-surface nanofibers and its controllable porosity, which can serve as a drug carrier [12,13]. Many natural and synthetic polymers have been used to obtain nanofiber-based scaffolds. The natural polymer and synthetic polymer mixture solution is the most promising approach that combines both polymer properties for electrospinning [14]. Silk sericin is an extracted protein obtained from Bombyx mori silk cocoons. Silk sericin (SS) consists of about 30% of the mass of cocoons, and is generally a waste product of the textile industry [15]. SS is a versatile biopolymer that has unique properties such as excellent biodegradability, good biocompatibility, oxygen and water-vapor permeability, and low immunogenicity. However, sericin alone does not possess the suitable mechanical properties required to prepare nanofibers due to its fragile nature. Therefore, blending sericin with other polymers is one of the best methods to improve its mechanical properties and broaden its potential applications [16,17]. Cellulose acetate, a cellulose derivative, is a water-soluble hydrogel polymer that can be quickly processed into membranes, films, and fibers [18]. Another polymer, polycaprolactone (PCL), is a synthetic polymer that possesses a variety of properties, such as biocompatibility, chemical stability, and excellent film-forming ability [8]. Both PCL and CA possess good mechanical properties, and are blended with natural biopolymers to increase the mechanical properties of the fiber. Therefore, SS has been combined with CA and PCL to improve nanofibers’ wound-healing and mechanical properties. Several novel wound dressings, including microsponges and polymeric nanofiber scaffolds, were developed for diabetic wound healing, and they proved to be a significant improvement in wound healing in diabetes-induced animal models [19]. However, a silk-sericin-based CA and PCL hybrid nanofibrous wound dressing loaded with natural bioactive molecules such as FA for diabetic wound healing has not been reported yet. Based on the fact, this study aimed to prepare and evaluate the in vitro characteristics and the in vivo diabetic-wound-healing potential of silk-sericin-based hybrid nanofibrous mats in a diabetes-induced rat model.

## 2. Materials and Methods

### 2.1. Materials 

Ferulic acid was obtained from P.C. Chem. Related. Ltd. (Mumbai, India). The Bombyx mori silkworm cocoons were received as a gift sample from the Lab of Sericulture, Department of Zoology, Babasaheb Bhimrao Ambedkar University (Lucknow, India). Cellulose acetate was purchased from GLR Innovations (New Delhi, India). Streptozotocin (STZ) was obtained from Sigma-Aldrich (Bangalore, India). Polycaprolactone was obtained from Thermo Fisher Scientific (Pune, India). Ketamine was obtained from Neon Lab Ltd. HaCaT cell lines were purchased from NCCS (Pune, India). All other chemicals and reagents used were of analytical and HPLC grade.

### 2.2. Extraction of Silk Sericin from Silk Cocoons

Silk cocoons weighing about 2.5 g were taken and cut into small pieces and immersed into a 250 mL solution consisting of 0.3% *w*/*v* sodium oleate and 0.2% *w*/*v* sodium carbonate. The process of degumming was performed at 100 °C for 1 h, and the solution was filtered to remove the degummed silk fibroin. After cooling the solution to room temperature, calcium chloride with 15% *w*/*v* was added, and the solution was continuously stirred for 3 h at room temperature and then centrifuged at 10,000 rpm for 20 min. The supernatant was dialyzed against distilled water for 3 days using a dialysis tube (Mol. cut-off; 8 kDa) and freeze-dried to obtain the silk sericin protein in a solid form. The precipitate was dried at 50 °C for overnight and analyzed further [20].

### 2.3. Fabrication of Silk Sericin Hybrid Nanofibers by Electrospinning 

The optimized concentrations for the electrospinning solutions has been mentioned in Table 1. The polymer solutions were prepared by mixing 12% *w*/*v* CA (cellulose acetate) in acetone and 12% *w*/*v* PCL (polycaprolactone) in DMF (dimethylformamide). FA 2% *w*/*v* was added to each polymer solution under continuous stirring. To obtain a uniform solution for electrospinning, a silk sericin solution of 8% *w*/*v* was mixed in both drug-loaded polymeric solutions and continuously stirred at room temperature to obtain a uniform dispersion. The FA-loaded silk sericin nanofibers were prepared by using the electrospinning process (E-spin Nanotech Pvt. Ltd. Kanpur, India). The solution (20 mL) was taken into a syringe (10 mL) having a stainless-steel needle with a 0.4 mm inner diameter. High-voltage power was supplied by connecting the positive electrode to the needle. A round cylindrical collector wrapped with aluminum foil was used to collect the FA-loaded silk sericin nanofibers, and was kept at a distance of 10 cm from the spinning electrode. The flow rate of the syringe pump was adjusted to 0.8 mL/h, and the rotating drum speed was kept at 300 rpm. The solutions were pumped at 10–25 kV of high electric voltage, depending on the formation of the Taylor cone once. The whole process was carried out under a defined humidity percentage (55%). 

### 2.4. Morphology of Nanofibers

Scanning electron microscopy (FESEM Quanta 200, Zeiss, Germany) was used for studying the morphology of the FA-loaded silk sericin nanofibers (SS-NF, FA-SS-PCL-NF, FA-SS-CA-NF) with an accelerating voltage at various magnifications. The surface of samples was made electrically conductive by placing it on specimen stubs for SEM and sputter-coated with Au–Pd for 1.5 min under an argon atmosphere. This evaluated the fibers’ morphology, pore size, diameter, and consistency. The average diameter and pore size were determined using ImageJ software.

### 2.5. Tensile Strength of Nanofibers

According to the ASTM standard test method, the mechanical properties of SS-NF, FA-SS-PCL-NF, and FA-SS-CA-NF were tested using a D882-02 single-column tensile tester (Zwick Roell Z010, Ulm, Germany). The nanofibrous samples were cut into the shape of a dog bone (10–30 mm), and their ends were attached to the gripping units of the tensile tester. A crosshead speed of 0.1 mm/s with a 4.5 kg load cell was applied to all nanofibers. The Young’s modulus (MPa) of all samples was calculated from the slope of the stress–strain curves. The following equations were used to calculate the elongation at break (*E_b_*) and ultimate tensile strength (UTS) [21]. where *L_max_* = extension at the moment of rupture (m), *L*_0_ = length of the sample, F_max_ = maximum load, and A = cross-sectional area of the sample.

$$E_{b}=\frac{L_{max}}{L_{0}}\times 100\;\;\;$$

$$\mathrm{UTS}=\frac{F_{\max}}{A}$$

### 2.6. Swelling Index

The swelling indexes of the FA-SS-PCL-NF and FA-SS-CA-NF were determined by incubating them in PBS at pH 7.4. The samples were dried at 50 °C under vacuum conditions. The nanofibers were cut into 1 × 1 cm^2^ pieces, and an electronic balance measured their dry weight (W_d_). After that, the samples were placed in PBS for 24 h. At determined intervals of time (0.5, 1, 2, 4, 8, 12, 16, 20, and 24 h), the samples were taken, and their wet weight (W_w_) was measured by placing them on filter paper for about 10 min to entrap moisture present in the nanofibers’ pores, then they were weighed [22]. The average swelling index was calculated by using the following equation:

$$\%\;\mathrm{Water}\;\mathrm{uptake}=\frac{W_{w}-W_{d}}{W_{d}}\times 100$$

where W_w_ = weight of the wet nanofiber scaffold and W_d_ = dried weight. 

A triplicate study was performed, and the average value was taken as the percentage of water uptake.

### 2.7. Biodegradability Assay

An in vitro biodegradation assay was conducted by incubating fabricated nanofibers in medium (PBS, pH 7.4) with lysozyme (104 unit/mL) at room temperature. Before that, they were dried in a vacuum condition for 24 h. Then, they were cut into 2 cm^2^ squares and weighed on an electronic balance. Each sample was placed in a shaker incubator at 37 °C in a separate test tube containing 10 mL of PBS; an 80 rpm speed was maintained, which simulated the body’s environment. The final weights of the nanofibers were measured every day before the medium was replaced. The samples were weighed again after 14 days. The assay was performed in triplicate [23]. The degradation index for the fabricated nanofibers was calculated by using the following equation:

$$\mathrm{WL}\%\;=\frac{\left(W_{i}-W_{f}\right)}{W_{i}}\times 100$$

where W_i_ = initial weight of the sample and W_f_ = final weight after degradation.

### 2.8. In Vitro Drug-Release Studies

The drug release from the prepared nanofibers was examined by cutting the mats in 1 cm^2^ pieces and incubating them in 10 mL PBS at pH 7.4 and 37 °C for 12 h at 100 rpm in a thermostatic shaking incubator. Then, 1 mL sample aliquots were withdrawn at determined time intervals and replaced with an equal amount of fresh PBS to maintain the sink condition [24]. The amount of FA release from the nanofibrous mats was examined spectrophotometrically at a wavelength of 216 nm at various time intervals in triplicate. The amount of drug released was plotted as the percentage (%) released against time, and the results were reported as the mean ± SD. 

### 2.9. Antimicrobial Activity

#### 2.9.1. Agar Disc Diffusion Method

The antimicrobial activity of the fabricated nanofibers against *Pseudomonas aeruginosa* (Gram-negative) and *Staphylococcus aureus* (Gram-positive) were measured using the agar disc diffusion method. The microbial strains *S. aureus* (ATCC 25923) and *P. aeruginosa* were grown in Tryptone soy broth (TSB) (Himedia LQ508) and Luria Bertani broth (LB) culture medium (Himedia M1245) respectively for 24 h at 37 °C at 200 rpm in an orbital shaker. The full-grown cultures of both the strains were spread using sterile cotton swabs on a tryptic soy agar plate and LB agar separately, in triplicate (Himedia M1968). Then, 6 mm wells were formed aseptically on an agar plate with a sterile cork borer. Nanofibrous discs of 5 mm diameter were punched and sterilized with UV for two hours [25]. Nanofibrous discs (FA-SS-PCL-NF, SS-NF, FA-SS-CA-NF), a placebo control (negative control), and vancomycin antibiotic discs (1µg/mL; positive control) were placed in the agar well plate under sterile conditions using sterile forceps. Subsequently, the plates were preserved at 4 °C for 1 h and then incubated aerobically for 24 h at 37 °C. The antibacterial activity was determined by the formed clear zones or microbial inhibition zones. The diameter of inhibition zones (DIZ) was measured and recorded precisely. The test was carried out in triplicate to calculate the average inhibition zone. The diameters of the inhibition zones around the well discs demonstrated the antimicrobial effects of the prepared nanofibers.

#### 2.9.2. Time-Kill Assay

A time-kill assay is the most robust method for determining a bactericidal effect. It is the appropriate method for the dynamic interaction between an antimicrobial substance and microbes, because there is a time- or concentration-dependent antimicrobial effect [26]. This assay examined the antimicrobial activities of the prepared nanofibrous formulations against *Staphylococcus aureus* and *Pseudomonas aeruginosa*. Briefly, nanofibers, a positive control (vancomycin 1 ug/mL), and a negative control (culture without drug) were added to 5 mL *S. aureus* and *Pseudomonas aeruginosa* culture suspensions in separate culture tubes (modified to 1 × 10^6^ CFUs/mL as a starting inoculum). Vancomycin and gentamycin were added at a concentration of 1/2-fold of the minimum inhibitory concentration (MIC). The culture tubes were incubated for 24 h at 37 °C in an orbital shaker at 150 rpm. The colony-forming units (CFUs) were determined at 0 h, 6 h, and 24 h after plating of appropriate dilutions on the tryptic soy agar plate. These plates were incubated at 37 °C in an incubator, and the CFUs/mL were measured. Decreases in the CFU counts with or without nanofibers were determined at 0 h, 6 h, and 24 h. The experiment was repeated three times to obtain the statistical significances among the control and the nanofibers [27].

#### 2.9.3. Biofilm Formation Assay

A biofilm assay was performed in triplicate as described elsewhere with minor modifications [28]. *S. aureus* ATCC 12598 was cultivated in tryptic soy broth without glucose (TSB-0 g) (17 g/L tryptone; 3 g/L soytone; 2.5 g/L K_2_HPO_4_.3H_2_O_5_ 5g/L NaCl) and supplemented with 1% NaCl and 0.5% glucose. *P. aeruginosa* ATCC 27853 was cultivated in tryptic soy broth (Himedia M011). Respective strains were cultivated for 24 h in an appropriate medium as described above and then diluted 100xin fresh medium prior to inoculation of 150 µL culture in a 96-well microtiter plate in triplicate. The nanofibrous mats loaded with FA were punched at 5 mm, sterilized with UV for 2 h, and incubated with the controls in triplicate wells. The microtiter plate was incubated for 24 h at 37 °C without shaking. The next day, the nanofibrous mats were removed, and the culture was discarded. Nonadherent cells were washed with sterile medium (PBS; pH 7.5), and 150 µL of 95% ethanol was added into all the wells to fix the biofilm for 2 min. The wells were washed three times with 150 µL PBS and stained with 150 µL 1% crystal violet (*w*/*v*) for 5 min. The wells were again washed three times with PBS and dried in air. A quantitative assessment of the biofilm formation was performed by reading the absorbance at 570 nm in a plate reader [29].

### 2.10. In Vitro Cell-Line Study

#### 2.10.1. Cell Culture and Treatment

Human keratinocytes (HaCaT) were grown in 75 cm^2^ flasks in full Dulbecco’s Modified Eagle’s medium (DMEM), which also contained 10% heat-inactivated fetal bovine serum and 1% L-glutamine. Detaching of cells from the flasks was done with an EDTA–trypsin solution, and cell counting was done with a hemocytometer and Trypan Blue staining.

#### 2.10.2. In Vitro Cytotoxicity (MTT Assay)

The MTT assay was a helpful tool for determining the in vitro cytotoxicity. The yellow tetrazolium salt in MTT was converted to purple formazan in the presence of phenazine methosulfate (PMS) by the dehydrogenase enzymes in live cells in this experiment. The amount of formazan produced was related to the number of living cells in the system. HaCaT were seeded at a density of 1 × 10^4^ cells per well (in 100 µL of DMEM media) and cultured for 24 h in a 96-well plate. The plates were incubated for 24 h after the medium was replaced with nanofiber samples. Following that, 10 mL of MTT reagent at a 5 mg/mL concentration was added to each well and incubated for 4 h. The purple formazan was dissolved by adding 100 µL dimethyl sulfoxide to each well, including the control (which underwent no treatment), gently swirling to thoroughly mix, and then allowing it to stand at room temperature for about 30 min. The absorbance was measured at 570 nm using a microplate reader [30,31]. 

#### 2.10.3. In Vitro Scratch Wound Assay

The cultured HaCat cells at a concentration of 1 × 10^5^ cells mL^−1^ in the 96-well plate were incubated at 37 °C for 24 h to become confluent. By using a 200 µL pipette tip, a gap was created in the cells, and debris was removed by using PBS twice. The FA-loaded nanofibers were placed in a cell strainer for direct release into the media. Then, a fresh 2 mL of medium was added to each well. The samples were imaged by an optical microscope at 0, 24, and 48 h to determine the cell migration [32]. 

### 2.11. In Vivo Wound Healing Activity

The ethical committee approved all the animals for in vivo experiments under approval number BBDNIIT/IAEC/2019/10/01 from BBDNIIT, Lucknow. Pathogen-free albino Wistar rats having weight of 150–200 g were selected. All animals were placed separately in pathogen-free conditions at a temperature of 23 ± 2 °C and humidity of 55 ± 5% with a 12 h light/12 h dark cycle. Animals were fed standard pellets and water. The rats were separated into four groups, and their dorsal surface hair was shaved. Previously, they were treated with streptozotocin (60 mg/kg) in cold citrate buffer (pH 4.5) intravenously to induce diabetes [33]. After 2–3 days passed, a blood glucose level ≥120 mg/dL was considered diabetic. The rats were anesthetized by injecting ketamine hydrochloride (50 mg/kg body weight). Single rounded full-thickness excisional wounds (8 mm) were created on the backside of each animal using a skin biopsy punch. After recovery from anesthesia, the formulations were directly applied to the wound sites in contact with the subcutaneous tissue. A Tegaderm^TM^ dressing was applied to the wounds at the same time to protect them from bacterial infections. Diabetic wound control (toxic control) was used in group I, placebo control was used in group II, and FA-SS-PCL-NF and FA-SS-CA-NF treatments were used in groups III and IV, respectively. The differences in the wound areas were observed at intervals of 0, 5, 9, and 14 days after treatment [34,35,36,37].

### 2.12. Histopathology

Animals from every group were sacrificed on the 5th, 9th, and 14th days for the histopathology study, and wound biopsies were taken. All skin samples were kept overnight in 10% paraformaldehyde, followed by ethanol dehydration, and then embedded in paraffin with a microtome. The tissue was cut into 10 mm thick sections and stained with hematoxylin and eosin, and various parameters were observed under a light microscope.

### 2.13. Statistical Analysis

All data were analyzed statistically, and the results are presented as the mean ± SD. The data were analyzed by one-way ANOVA, and the differences among all group means were evaluated using an unpaired, two-sided Student’s *t*-test. Significant differences were considered as a *p*-value less than 0.05, while those with a *p*-value less than 0.01 were considered highly significant.

## 3. Results and Discussion

### 3.1. Surface Morphology

**Figure 1:**
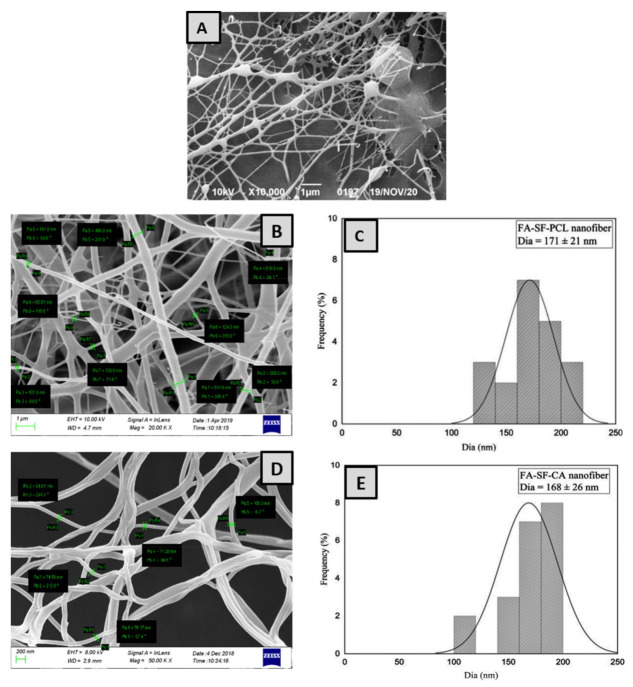
SEM images of nanofibers and their average diameters: (**A**) SS-NF; (**B**,**C**) FA-SS-PCL-NF; (**D**,**E**) FA-SS-CA-NF.

The electrospinning technique was successfully used to prepare silk-sericin-based hybrid nanofibrous mats loaded with FA for diabetic wound healing. The formulation parameters, such as concentration of the polymer, applied voltage, distance between the pump and collector, and flow rate were optimized and chosen during the electrospinning process. SEM assessment demonstrated the surface morphology, nanofiber diameter, and homogeneity of optimized nanofibers. The nanofibers prepared with silk sericin alone were irregular, with a rough surface and beads in between the fibers, as shown in Figure 1A. On the other hand, the nanofibers prepared by blending silk sericin with PCL and CA showed a fine, smooth, flexible interconnected structured fiber, as shown in Figure 1B,D. The results demonstrated that the mechanical properties of the silk sericin were tremendously improved when blended with CA and PCL polymers. The average diameters of the hybrid nanofibers (FA-SS-PCL-NF and FA-SS-CA-NF) were found to be 171 ± 21 nm and 168 ± 26 nm, respectively, as seen in Figure 1C,E. The hybrid nanofibers prepared by the electrospinning process in this study were uniformly oriented with interconnecting pores and a weblike porous structure, which was essential for oxygen exchange, fluids, nutrients, fibroblasts, cell adhesion, and cell attachment. The findings showed that the developed hybrid nanofibers exhibited all the desired physical and structural properties for rapid wound healing [38,39].

### 3.2. Mechanical Properties

**Figure 2:**
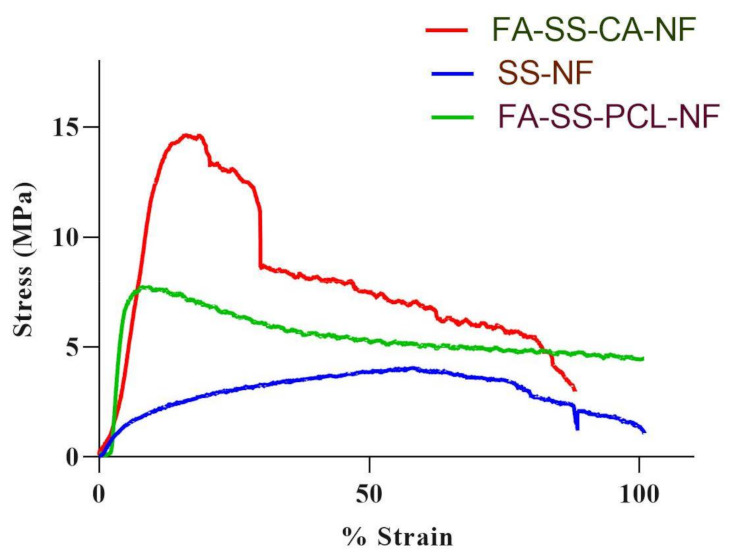
Graph of tensile strength of nanofibrous formulations.

As described in earlier research, the mechanical strength was measured to determine the force required to withstand the maximum stress per unit area. Mechanical properties are necessary for nanofibers, because their matrix must possess sufficient mechanical strength to effectively repair the tissue [40]. A regular fragile crack with poor rigidity was observed in a pure silk sericin nanofiber with a poor tensile strength of 4.04 MPa, as shown in Figure 2. The tensile strength was improved sufficiently when sericin was mixed with CA and PCL, and the tensile strengths of the nanofiber formulations FA-SS-PCL-NF and FA-SS-CA-NF were found to be to 7.73 MPa and 14.65 MPa, respectively (Table 2). An increase in strain will increase the stress up to a maximum, but at certain interval, the stress will begin decreasing, and the nanofibers will break.

### 3.3. Swelling Index

**Figure 3:**
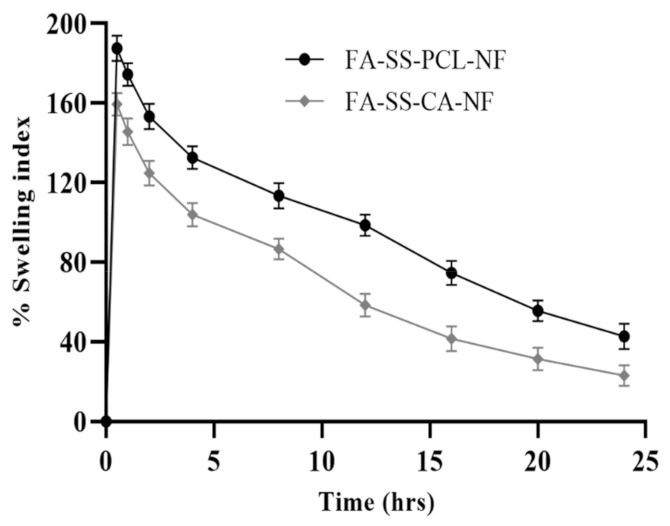
Swelling index (%) of FA-SS-PCL-NF- and FA-SS-CA-NF-optimized nanofibrous formulations in PBS at pH 7.4 (mean ± S.D.).

The swelling index of the nanofibers was measured based on their water absorption capacity or the number of wound exudates that could be easily absorbed when applied as dressings. Formulations with PCL (FA-SS-PCL-NF) and CA (FA-SS-CA-NF) both possessed hydrophobic, and initially, there was a sharp increase in the absorption and retention capacity of the nanofibers; after that, there was a slight decrease in the water-retention capacity of both types of nanofibers. Silk sericin and PCL nanofibers showed a higher water-retention capacity than silk sericin with CA nanofibers, because the CA was more hydrophobic than the PCL. The maximum swelling of the FA-SS-PCL-NF batch was found to be 187 ± 6.4% after half an hour, whereas the maximum swelling of the FA-SS-CA-NF batch was found to be 159.42 ± 5.6% after half an hour (Figure 3). Further, the water-uptake capacity in both the batches gradually decreased up to 24 h. This could have been due to the shielding effect of the hydroxyl and carboxyl groups and the tight hydrogen bond used in polymers. The swelling index of the various polymer nanofibers is shown in Figure 3.

### 3.4. Biodegradability Assay

**Figure 4:**
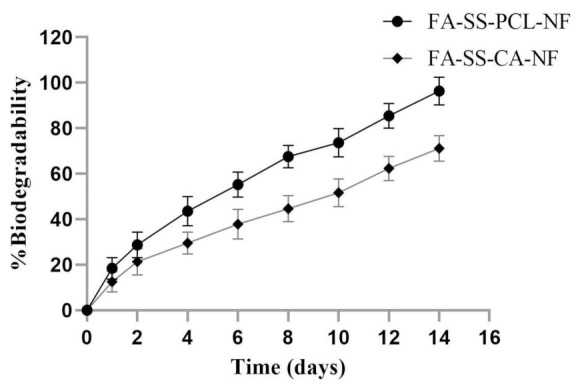
Percentage biodegradability of optimized nanofibrous formulations FA-SS-PCL-NF and FA-SS-CA-NF in PBS at pH7.4 (mean ± S.D.).

The in vitro biodegradation test was conducted by immersing the samples in PBS, and the weight loss at various time intervals was assessed as shown in Figure 4. The results showed that formulation batch FA-SS-PCL-NF had degraded by almost 95.63% on day 14 in PBS at pH 7.4, while the nanofiber batch FA-SS-CA-NF degraded by 72.42% after 14 days. The difference was due to the hydrophobic nature of both formulations; the higher the hydrophobicity, the slower the degradation. Cellulose acetate was more hydrophobic than PCL polymer; that is why the formulation with CA degraded slowly and lasted more than 14 days, and the formulation with PCL degraded faster, by almost 95.63% on day 14 [8]. So, this demonstrated that the formulation with PCL biodegraded faster than the formulation with CA.

### 3.5. In Vitro Drug Release Studies

**Figure 5:**
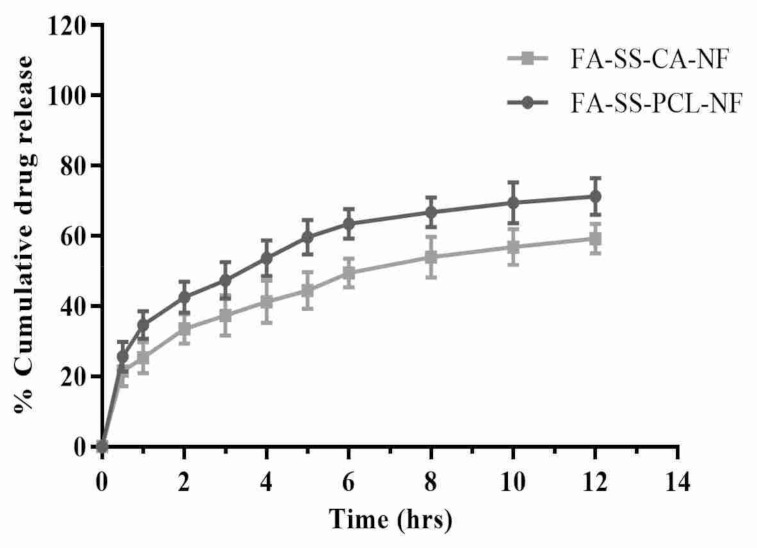
In vitro drug release of FA-SS-PCL-NF and FA-SS-CA-NF nanofibrous formulations in PBS at pH7.4 (mean ± S.D.).

The drug release of the fabricated nanofiber scaffolds is shown in Figure 5. The drug-loaded nanofibrous formulation showed an initial release (21–35%) in the first hour due to degradation in the polymer layer, followed by a delayed-release pattern due to diffusion of the drug. The maximum drug releases from the FA-SS-PCL-NF and FA-SS-CA-NF were 71.23% and 59.24%, respectively, after 12 h (Table 3). These results showed that the nanofibers produced could effectively deliver the drug to the wound site over a more extended period. The rapid burst release of these dosage forms assisted in maintaining the effective concentration as adequate to provide the optimum pharmacological response in a short duration, followed by a sustained release that sustained an equivalent therapeutic response over a prolonged period of time [22,41].

### 3.6. Antimicrobial Study

**Figure 6:**
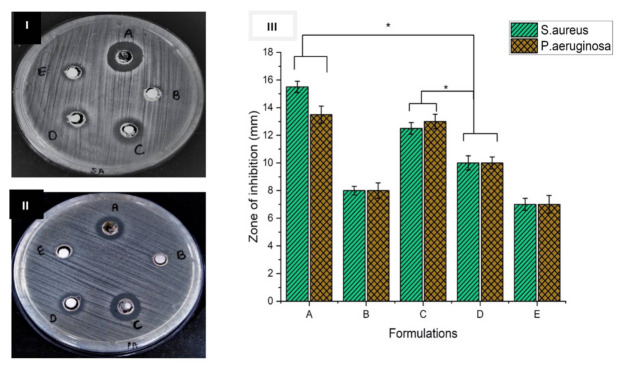
Inhibition zones of the nanofiber discs: (**A**) FA-SS-PCL-NF; (**B**) placebo; (**C**) FA-SS-CA-NF; (**D**): +ve control; (**E**) SS-NF against (**I**) Staphylococcus aureus and (**II**) Pseudomonas aeruginosa. (**III**) Bar graph of disc diffusion assay results. * Significant differences between control and the formulations corresponding to each bacteria.

The antimicrobial activity is an essential area of study of nanofibers for ideal wound healing activity. As they were to be used in wound healing and tissue regeneration, the FA-loaded nanofibers also needed to maintain the ability to protect against microbial species. The antimicrobial study was conducted using an agar disc diffusion method against *Staphylococcus aureus* and *Pseudomonas aeruginosa*. The zone of inhibition formation was an indication of antimicrobial activity. Figure 6 illustrates the results for the antibacterial activities of the FA-loaded nanofibers [42]. The results obtained from the disc diffusion assay against *S. aureus* and *P. aeruginosa* showed that the silk-sericin-based FA-loaded nanofibers produced a higher zone of inhibition against both the bacterial strains (Figure 6I(A,C),II(A,C)). This clearly indicated that they possessed a better antimicrobial property. If we increased the concentration of the drug in the formulation of the nanofibrous scaffolds, the inhibition zone would increase [25]. Figure 6III shows a bar graph of the zones formed by all the formulations.

### 3.7. Time-Kill Assay Results

**Figure 7:**
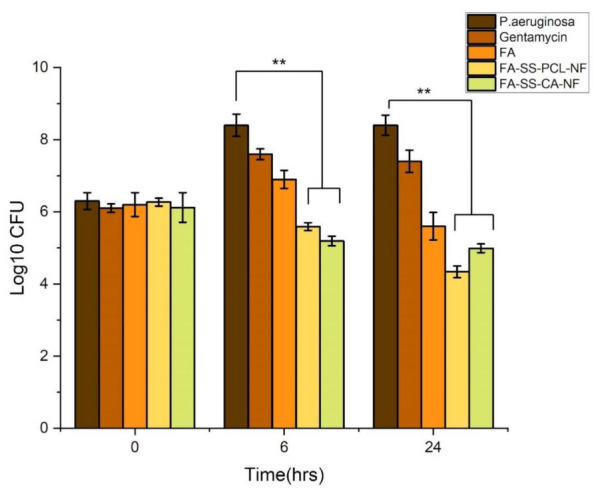
Bar graph of time-kill assay results against Pseudomonas aeruginosa. The error bars indicate the mean ± S.D. ** Significant differences between control, drug, and the formulations corresponding to bacteria.

**Figure 8:**
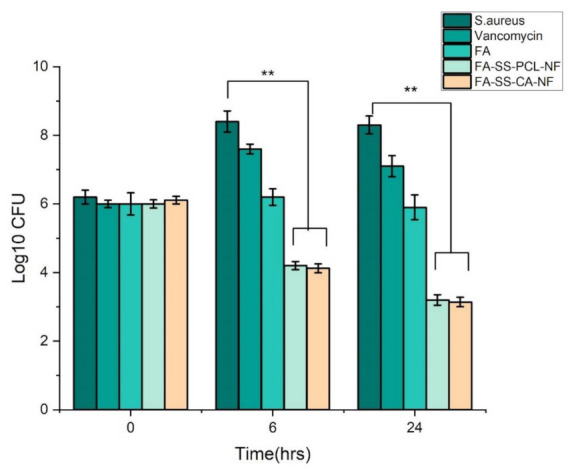
Bar graph of time-kill assay results against Staphylococcus aureus. The error bars indicate the mean ± S.D. ** represent the significant differences between control, drug, and the formulations corresponding to bacteria.

Determination of the antibacterial activity in the presence of nanofibrous mats was time-dependent, as shown in Figure 7 and Figure 8. After incubation of the nanofibrous mats with *S. aureus* and *Pseudomonas aeruginosa* cultures at 0, 6, and 24 h, CFU counts significantly decreased compared to the control *S. aureus* and *Pseudomonas aeruginosa* cultures without any drug. Interestingly, FA-SS-PCL-NF and FA-SS-CA-NF nanofibers showed a higher reduction in CFU counts than the FA drug alone, suggesting the nanofibrous formulation increased the antibacterial activity of the drug (Figure 7 and Figure 8) [26,27]. Furthermore, the nanofibrous formulation increased the antimicrobial activities with time until 24 h due to the sustained-release characteristics of the nanofibrous formulations, as shown in Figure 7.

### 3.8. Biofilm Assay

**Figure 9:**
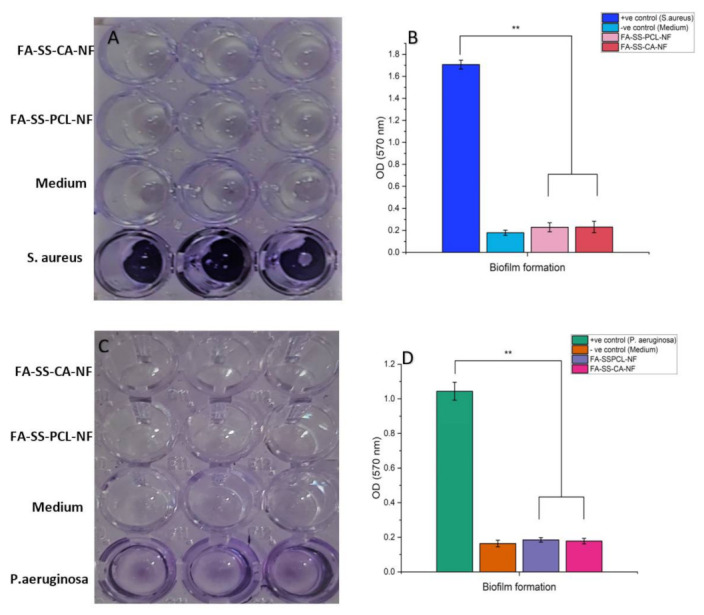
Quantitative estimation of biofilm formed in the presence of nanofibrous mats loaded with FA, medium control, or test bacterium. (**A**,**C**) Microplate wells before absorbance reading. (**B**,**D**) Average absorbance of triplicate. ** represent the significant differences between positive control and the formulations.

In the biofilm formation assay, we found that *S. aureus* and *P. aeruginosa* produced biofilm in the TSB-0 g supplemented with 0.5% glucose and the 1% NaCl and TSB medium, respectively (Figure 9). The culture incubated with nanofibrous mats loaded with FA could not produce a biofilm as compared to the control culture. This suggested that the developed FA-SS-PCL-NF and FA-SS-CA-NF nanofibers produced a significant inhibitory effect on biofilm formation by both Gram-positive (*S. aureus*) and Gram-negative (*P. aeruginosa*) bacteria.

### 3.9. In Vitro Cytotoxicity (MTT Assay)

**Figure 10:**
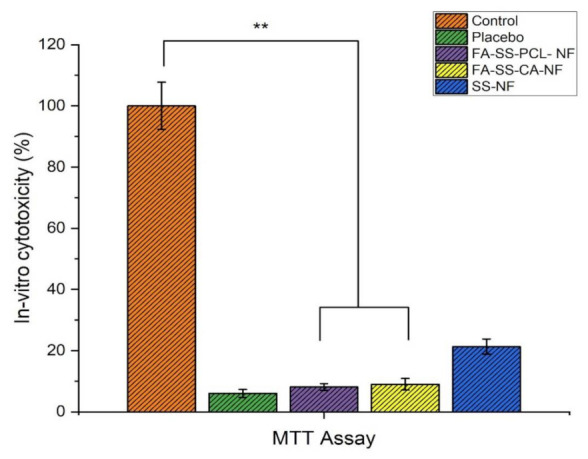
Quantitative assay of in vitro cytotoxicity of all formulations by MTT on HaCaT cells (mean ± S.D). ** Significant differences between control and the formulations.

The MTT assay estimates the number of viable cells by lowering the MTT tetrazolium compound by viable active cells, reflecting normal mitochondrial function, whereas Trypan Blue staining is predicated on the assumption that healthy cells have intact cell membranes that are not stained [30]. Over 24 h of cultivation, in vitro cytotoxicity tests revealed a lower cytotoxicity. Furthermore, the number of cells rose constantly throughout 24 h of growth, demonstrating that all nanofibers were less toxic, according to the optical density of the MTT assay. As shown in Figure 10, the number of viable and dead cells were counted, and the mean SD cell viability % of HaCaT cells was calculated. Cell viability of more than 90% was deemed nontoxic. The MTT assay revealed a cell viability of above 97%. These findings provided scientific proof that the FA-loaded nanofibrous membrane was non-toxic to HaCaT cells. In addition, findings from an in vitro cytotoxicity assay showed that FA-loaded nanofibers were biocompatible and nontoxic to HaCaT cells, with more significant growth rates [9]. 

### 3.10. In Vitro Scratch Wound Assay

**Figure 11:**
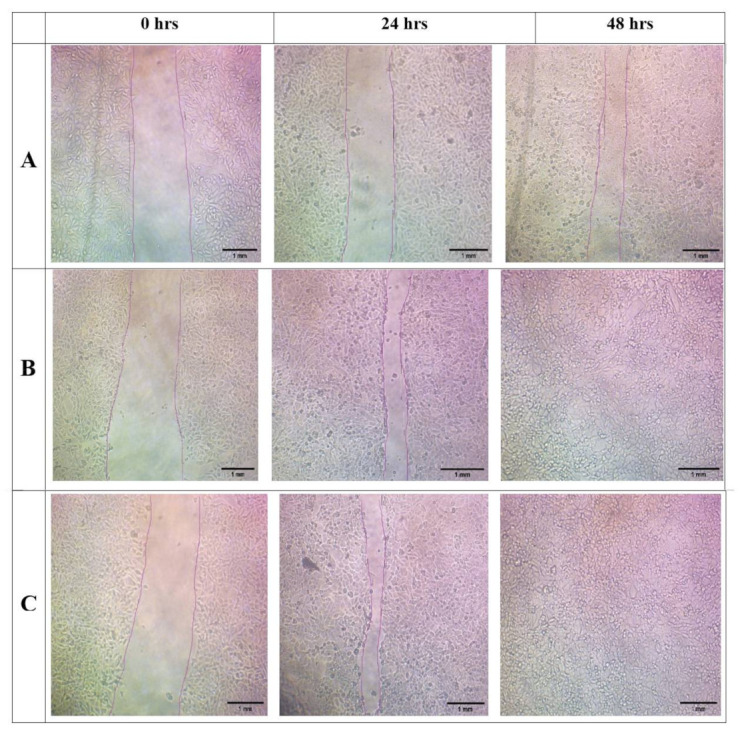
Images of in vitro scratch assay of formulations of (**A**) (control), (**B**) (FA-SS-PCL-NF), and (**C**) (FA-SS-CA-NF) at 0, 24, and 48 h (scale bar = 1 mm).

**Figure 12:**
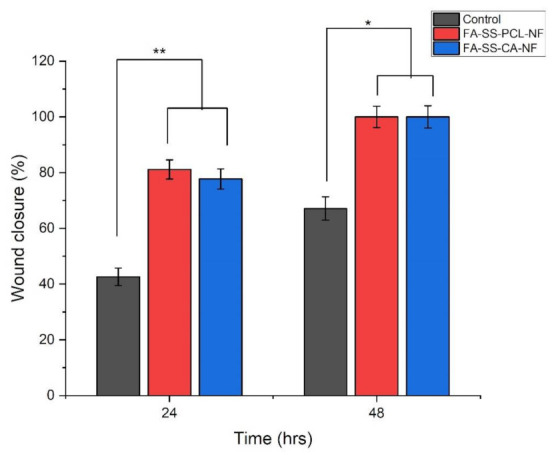
Graphical representation of % wound closure of in vitro wound assay at 24 and 48 h (mean ± S.D). * and ** represent the significant differences between the control and the formulations.

In the reepithelization process, keratinocytes migrate to cover the wound and proliferate to form a dense epithelium [43]. To evaluate the effects of FA-loaded nanofibers on keratinocyte migrations, an in vitro scratch wound assay was performed, and the results are shown in Figure 11. The distance between scratch wounds was similar across all the samples at 0 h. After 24 h of incubation upon scratching, FA-loaded nanofibers promoted the moderate migration of cells as compared to the control. The highest performance was observed after 48 h in HaCaT cells incubated with FA-SS-PCL-NF and FA-SS-CA-NF, closing a maximum of the scratched wound. Thus, from the images, we found that the prepared nanofibers promoted the keratinocytes to migrate toward the wound and fill the gap to increase the wound contraction. The percentage of wound contraction at 24 and 48 h is shown in Figure 12. The FA-SS-PCL-NF showed 81% wound contraction at 24 h and 100% at 48 h, while the FA-SS-PCL-NF showed 77% wound contraction at 24 h and 100% at 48 h. The control showed 42% wound contraction at 24 h and 67% at 48 h. So, it was concluded that both the nanofibers (FA-SS-PCL-NF and FA-SS-CA-NF) showed significant wound contraction in comparison to the control.

### 3.11. Weight Variation in Diabetes-Induced Animals 

**Figure 13:**
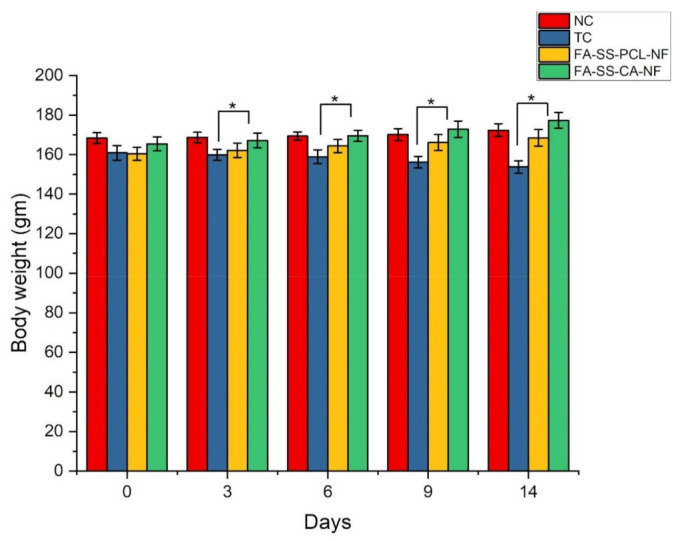
Quantitative result of the effect of diabetes on body weight in diabetic rats (mean ± S.D). * Significant differences between control and the formulations.

Streptozotocin 60 mg/kg intravenously was used to induce diabetes. After inducing diabetes in all animal groups, the mean increase/decrease in body weight was noted and recorded for all animal groups at different time intervals. A significant decrease in the mean body weight of the toxic animal group was observed on days 0, 3, 6, 9, and 14 compared to the standard control and nanofiber-treated groups, suggesting that diabetes was adequately induced in the animal group (Table 4). However, fewer changes were observed in either treatment group. The blood sugar level and body weight were measured before and after the induction of diabetes. The average body weight of the diabetes-induced rats is shown in Figure 13. The blood sugar level increased and the body weight decreased significantly in the toxic control, while the treatment group showed a lower reduction in body weight [44].

### 3.12. In Vivo Wound-Healing Study

**Figure 14:**
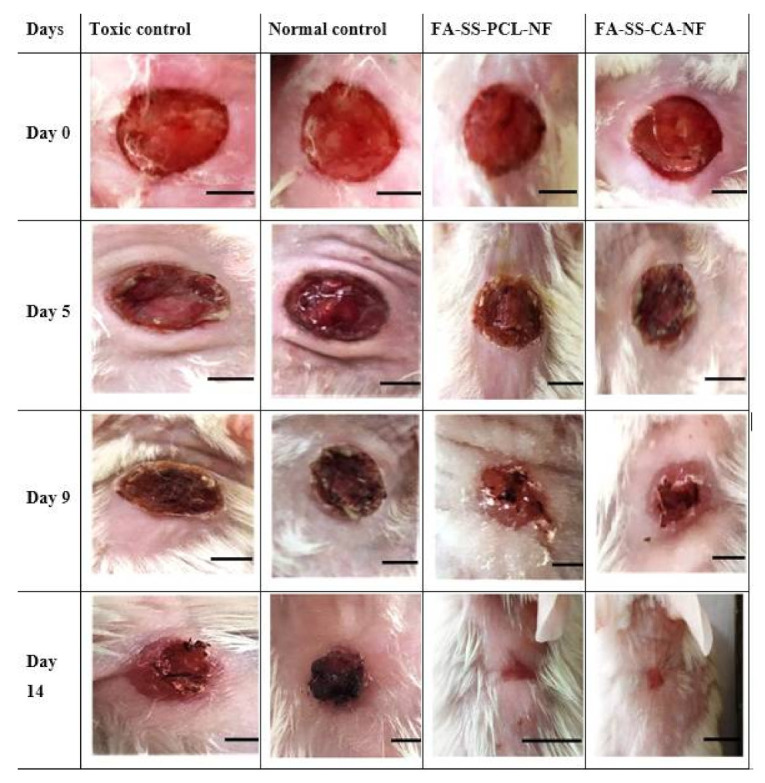
Images of wound healing in streptozotocin-induced diabetic rats on days 0, 5, 9, and 14 day after treating animals with TC (toxic control), NC (normal control), or formulation batches FA-SS-PCL-NF and FA-SS-CA-NF (scale bar = 4 mm).

An in vivo wound-healing study of the fabricated nanofiber scaffolds was performed by the excision wound method and studied in Streptozotocin-induced diabetic rats at different time intervals. The wounds were photographed on days 0, 5, 9, and 14 from the same height/distance. The wounds were scored, and the wound areas were measured on days 0, 5, 9, and 14 to determine the wound-healing potential of the nanofiber formulations. Figure 14 shows that the ferulic acid-loaded nanofibers based on silk made from PCL and CA had a better wound-healing effect than the control groups. This was due to the wound-healing and antioxidant properties of ferulic acid, which blended with the nanofibers [36]. Ferulic acid also accelerated wound healing and promoted skin regeneration [45]. Furthermore, this also was due to the webbed structure of the nanofibers, which had a high surface area and contributed to more significant absorption of the wound exudates. In addition, the porous networklike nanofiber structure mimicked the extracellular matrix (ECM) of the skin. It promoted adequate cellular respiration and optimal water and oxygen permeability, and maintained a moist environment at the wound site, which was essential for potential wound healing. [46].

**Figure 15:**
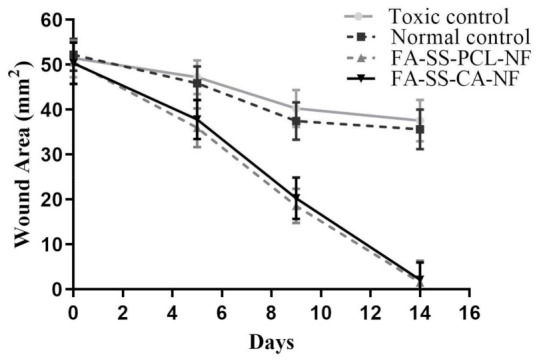
Wound-healing rate for normal control, toxic control, FA-SS-PCL-NF, and FA-SS-CA-NF (mean ± S.D.).

The wound-healing potential of all the nanofibers was evaluated by measurement of the wound areas in mm^2^ for all the groups on days 0, 5, 9, and 14 (Figure 15). On day 0, the wound areas for the toxic control, standard control, FA-SS-PCL-NF, and FA-SS-CA-NF were 51.49, 52.24, 50.56 and 50.32 mm^2^, respectively. On day 5, the wound areas for the toxic control and normal control were 47.24 and 45.86 mm^2^, respectively, which were higher than those of the FA-SS-PCL-NF (35.95) and FA-SS-CA-NF (37.83 mm^2^), which showed smaller wound areas as compared to the control groups. Similarly, on day 9, the FA-SS-PCL-NF and FA-SS-CA-NF had wound areas of 18.56 mm^2^ and 20.27 mm^2^, respectively, which showed a higher rate of wound healing than that of the control groups, as the wound areas for control groups were larger. They were found to be 40.26 mm^2^ (toxic control) and 37.49 mm^2^ (normal control), which were significantly (*p* < 0.01) higher than those of the FA-SS-PCL-NF and FA-SS-CA-NF groups. The maximum wound healing and the minimum wound area were achieved on day 14 for the groups treated with FA-SS-PCL-NF and FA-SS-CA-NF, as shown in Figure 15, and were found to be 1.52 and 2.12 mm^2^, respectively; whereas for the control groups, it was found to be 37.59 mm^2^ for the toxic control group and 35.63 mm^2^ for the standard control group [47].

### 3.13. Histopathology

**Figure 16:**
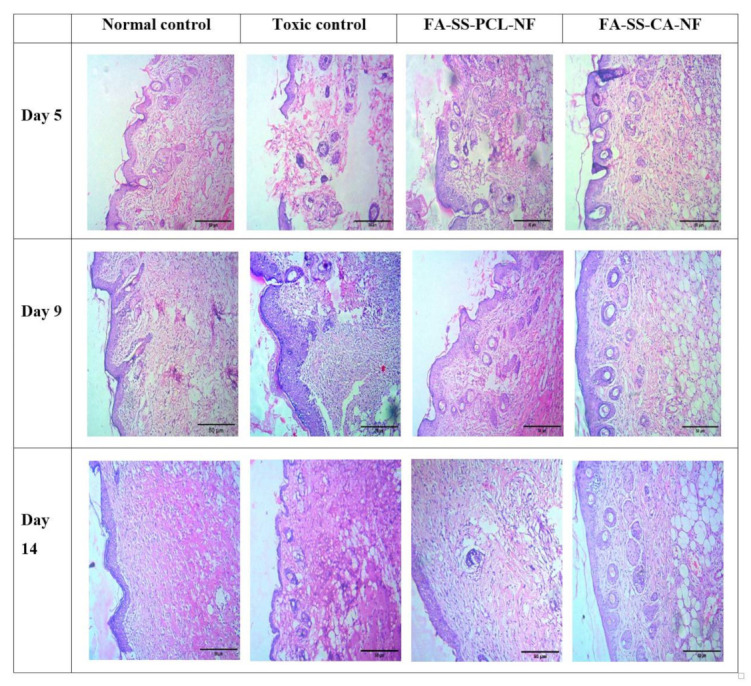
Histopathological images of normal control, toxic control, FA-SS-PCL-NF, and FA-SS-CA-NF (scale bar = 50 µm).

The histological images (showing staining with H&E) in Figure 16 showed that the toxic group had a slower cell arrangement than the treatment groups. In the treatment groups, the cells were assembled, and the healing process occurred. The cells were assembled with reduced inflammatory cells and increased fibroblasts, which signified the beginning of the proliferative phase, and the healing took place due to regeneration of the epithelial layer. Furthermore, the observation of fibrin and fibroblasts indicated collagen formation [48,49]. The results showed that the modeling and wound-healing maturation stage were completed on the 14th day in the nanofiber-treated group. 

## 4. Conclusions

Impaired wound healing is considered one of the major complications in diabetic patients, leading to amputation. Silk-sericin-based hybrid nanofibers loaded with ferulic acid were successfully prepared using the electrospinning technique and different proportions of PCL and CA. The results of the SEM evaluation confirmed the fine, smooth, and flexible interconnected nanostructured fiber. The fabricated nanofibers also exhibited a desirable mechanical strength and biodegradability, and maintained adequate moisture for better wound healing. The in vitro drug-release studies confirmed the sustained-release property of the nanofibers for a prolonged period. Furthermore, the histological images of the wound also demonstrated the healing properties of the nanofibers. In addition, the in vivo studies in the diabetes-induced rat models found significantly more wound healing than in the control groups. The antimicrobial studies showed that the prepared nanofibers possessed good antimicrobial and antibiofilm activities against *Staphylococcus aureus* and *Pseudomonas aeruginosa.* In-vitro cell line studies revealed that the nanofiber formulation had a much less toxic effect on the HaCat cell lines, and the cell migration assay results indicated the increased migration of cells to the wound site to fill the gap. Furthermore, the histology study confirmed epithelial layer regeneration and collagen formation at the wound site. Based on these results, we be concluded that the developed nanofiber formulation had excellent wound-healing properties in the diabetes-induced rats. A better scale-up ability of this formulation could lead to future commercialization, and it could be an effective tool for potential wound-healing applications in diabetic persons. Further long-term studies should be pursued in this direction.

## Figures and Tables

**Table 1:** Optimized concentrationof polymers and drug solution for electrospinning.

Formulation	SS	PCL	CA	FA
SS-NF	8%	-	-	-
FA-SS-PCL-NF	8%	12%	-	2%
FA-SS-CA-NF	8%	-	12%	2%

**Table 2:** Average tensile strength of formulations.

S. No.	Formulations	Strength (MPa)
1.	SS-NF	4.04 ± 0.013
2.	FA-SS-PCL-NF	7.73 ± 0.204
3.	FA-SS-CA-NF	14.65 ± 0.159

**Table 3:** Cumulative (%) drug release data of optimized formulations (mean ± S.D.).

Time (h)	FA-SS-PCL-NF	FA-SS-CA-NF
0	0	0
0.5	25.67 ± 4.21	21.56 ± 4.34
1	34.65 ± 3.95	25.34 ± 4.45
2	42.56 ± 4.45	33.56 ± 4.23
3	47.34 ± 5.21	37.34 ± 5.67
4	53.68 ± 5.12	41.23 ± 5.98
5	59.65 ± 4.89	44.45 ± 5.23
6	63.45 ± 4.21	49.46 ± 4.12
8	66.78 ± 4.32	53.93 ± 5.85
10	69.45 ± 5.78	56.83 ± 5.12
12	71.24 ± 5.23	59.23 ± 4.18

**Table 4:** Effect of diabetes on the average body weight of diabetes-induced rats before and after the treatment with formulations (FA-SS-PCL-NF and FA-SS-CA-NF) at a different time intervals (days 0, 3, 6, 9, and 14) (mean ± SD; *n* = 6) NC: normal control; TC: toxic control.

Groups	Body Weight (gm) (before Inducing Diabetes)	Body Weight (gm) (after Inducing Diabetes)				
		0 Day	3rd Day	6th Day	9th Day	14th Day
NC	170 ± 2.11	168.4 ± 2.71	168.7 ± 2.66	169.4 ± 2.11	170.2 ± 3.01	172.4 ± 3.21
TC	172 ± 2.63	160.9 ± 3.65	159.9 ± 2.76	158.9 ± 3.46	156.2 ± 2.94	153.8 ± 3.12
FA-SS-PCL-NF	171 ± 2.47	160.4 ± 3.25	162.2 ± 3.66	164.4 ± 3.32	166.2 ± 4.01	169.5 ± 4.21
FA-SS-CA-NF	175 ± 3.12	165.5 ± 3.51	167.2 ± 3.71	169.5 ± 2.83	172.9 ± 4.11	177.4 ± 4.03

## Data Availability

Data sharing contains in this article.
